# Metamaterials with index ellipsoids at arbitrary **k**-points

**DOI:** 10.1038/s41467-018-04490-4

**Published:** 2018-05-25

**Authors:** Wen-Jie Chen, Bo Hou, Zhao-Qing Zhang, John B. Pendry, C. T. Chan

**Affiliations:** 1 Department of Physics and the Institute for Advanced Study, The Hong Kong University of Science and Technology, Hong Kong, China; 2 College of Physics, Optoelectronics and Energy, and Collaborative Innovation Center of Suzhou Nano Science and Technology, Soochow University, Suzhou, China; 3 Key Laboratory of Modern Optical Technologies of Ministry of Education & Key Lab of Advanced Optical Manufacturing Technologies of Jiangsu Province, Suzhou, China; 4 Condensed Matter Theory Group, Physics Department, Imperial College, London, SW7 2AZ UK

## Abstract

Propagation behaviors of electromagnetic waves are governed by the equifrequency surface of the medium. Up to now, ordinary materials, including the medium exist in nature and the man-made metamaterials, always have an equifrequency surface (ellipsoid or hyperboloid) centered at zero **k**-point. Here we propose a new type of metamaterial possessing multiple index ellipsoids centered at arbitrary nonzero **k**-points. Their locations in momentum space are determined by the connectivity of a set of interpenetrating metallic scaffolds, whereas the group velocities of the modes are determined by the geometrical details. Such system is a new class of metamaterial whose properties arise from global connectivity and hence can have broadband functionality in applications such as negative refraction, orientation-dependent coupling effect, and cavity without walls, and they are fundamentally different from ordinary resonant metamaterials that are inherently bandwidth limited. We perform microwave experiments to confirm our findings.

## Abstract (web summary)

Ordinary materials have their index ellipsoids centered at zero momentum. The authors propose a metamaterial of interpenetrating wire meshes whose connectivity can be used to control the number and position of index ellipsoids at arbitrary nonzero **k**-points. This could provide a new platform for broadband functionality.

## Introduction

Metamaterials^1–6^ have been proposed to realize exotic effective permittivity and permeability that are not found in nature and thereby realizing interesting phenomena such as negative refraction^7–10^ and electromagnetic cloaking^11–13^. Most metamaterials derive their properties from built-in subwavelength resonant structures^1,2^ and their effective constitutive parameters do not rely on the arrangement of the resonant structures. The equifrequency surface typically forms an index ellipsoid (double positive/negative medium) or hyperboloid (hyperbolic medium^14^) centered at **k** = 0. In the quasistatic limit, effective permittivity and permeability are normally well defined, and we expect a linear dispersion at low frequencies near **k** = 0, i.e., *ω* → 0 as **k** → 0.

We propose a new type of wire metamaterial^1,15–40^ possessing one or more index ellipsoids centered at nonzero **k**-points, with linear bands that go to zero frequency at some **k** ≠ 0. The existence of the quasistatic modes at nonzero **k**-points can be understood by solving Poisson’s equation and can be viewed as the shifting of the light cone by a synthetic gauge potential induced by the twisting in network. The number of ellipsoids and their positions within the Brillouin zone can be controlled by changing the connectivity of the wire meshes. This gives us a new degree of freedom to tailor the electromagnetic response of metamaterials that are based on connectivity rather than resonance, leading to unusual wave propagation behaviors.

## Results

### Quasistatic modes

**Fig. 1:**
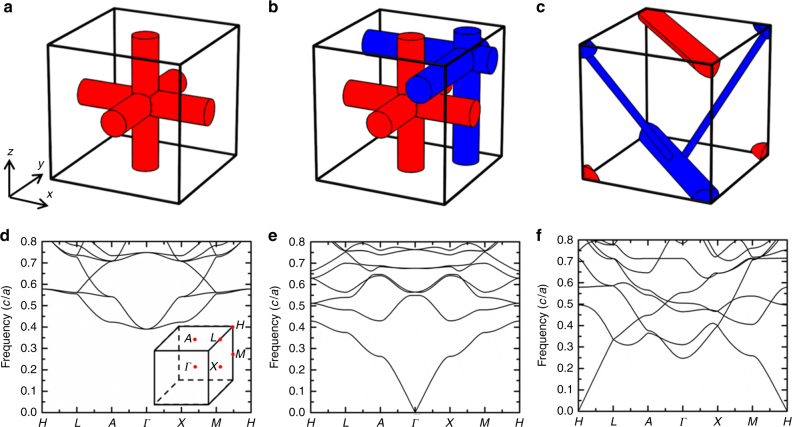
Three types of wire mesh metamaterials. All have a simple cubic lattice with lattice constants of *a*. **a** Unit cell composing of a single wire mesh. **b** Unit cell containing two wire meshes. Compared with **a**, another wire mesh (blue) is added, whose node is centered at $$(\begin{array}{*{20}{c}} {0.3a} & {0.3a} & {0.3a} \end{array})$$. **c** A more complex metamaterial composing of two wire meshes. All of the metallic wires (both red and blue) in **a**–**c** are assumed to be perfect metal. Their different colors indicate that the two meshes have independent potentials. **d**–**f** Corresponding band structures. The single wire mesh structure **d** has no eigenmode at zero frequency, while the two types of double wire mesh structures have a light cone at Brillouin zone center **e** or corner **f**. The inset in **d** shows the Brillouin zone of a simple cubic lattice

To illustrate our idea, we start by considering three different types of metallic wire scaffold structures as shown in Fig. 1a–c. The wire meshes, assumed to be perfect metal, are arranged in simple cubic lattices with lattice constants *a*. The band structure in Fig. 1d corresponds to the simple cubic wire mesh shown in Fig. 1a. It has a cutoff frequency of 0.39(*c*/*a*). The double wire mesh shown in Fig. 1b is formed by two copies of wire meshes shown in Fig. 1a. Intuitively, low-frequency waves should be more difficult to pass as the metallic volume ratio is doubled, and hence the low-frequency gap should persist. However, the calculated band structure shown in Fig. 1e shows that the double wire mesh has a light cone at **k** = 0. These disconnected wire structures^27–30^ are called non-Maxwellian media^28^. Disconnected crossed wire arrays^31–35^ can exhibit anomalous refraction due to its double hyperbolic equifrequency contours centered at **k** = 0. The band structure shown in Fig. 1f corresponds to a more complex double wire mesh structure shown in Fig. 1c. It distinguishes from other wire structures, because it has a “light cone” emerging from the *H* point. We note that the bands in the light cones shown in Fig. 1e,f are singly degenerate and they are quasi-longitudinal modes.

The existence of these quasi-longitudinal bands can be understood by considering the quasistatic limit (*ω* → 0) where the Maxwell’s equation reduces to Poisson’s equation $\nabla ^2{\it{\varphi }} = 0$ and ${\mathbf{E}} = - \nabla {\it{\varphi }}$, with *φ* representing the quasistatic potential. We first consider a single wire mesh (Fig. 1a) with a wire radius of 0.1*a*. Once the potential *φ*_1_ of the wire mesh is fixed, the uniqueness theorem implies that the system has a unique solution of the electric field, which can only be zero in the whole space. The system in Fig. 1a has no eigenmode at zero frequency and hence a cutoff frequency.

Next, we consider the double mesh structure in Fig. 1b, where we add another identical copy of the wire mesh (blue), shifted from the original (red) by a displacement of (0.3*a* 0.3*a* 0.3*a*). As the red and blue meshes do not touch each other, they have independent quasistatic potentials, *φ*_1_ and *φ*_2_, allowing for a nontrivial solution when (*φ*_2_−*φ*_1_) is nonzero. The system must have one zero-frequency mode lying somewhere (with Bloch **k** = (*k*_*x*_ *k*_*y*_ k_*z*_) in the Brillouin zone. We note that we can apply Bloch theorem to the potential differences. If the left end of the red (blue) wire has a potential *φ*_1_ (*φ*_2_) and their potential difference is (*φ*_2_−*φ*_1_), then the potential difference between their right ends should be $\left( {{\it{\varphi }}_2 - {\it{\varphi }}_1} \right)e^{ik_xa}$ due to periodicity. As the left and the right end are connected by a perfect metallic wire, their potentials are equal; therefore, $\left( {{\it{\varphi }}_2 - {\it{\varphi }}_1} \right) = \left( {{\it{\varphi }}_2 - {\it{\varphi }}_1} \right)e^{ik_xa}$. Likewise, we also have $\left( {{\it{\varphi }}_2 - {\it{\varphi }}_1} \right) = \left( {{\it{\varphi }}_2 - {\it{\varphi }}_1} \right)e^{ik_ya}$ and $\left( {{\it{\varphi }}_2 - {\it{\varphi }}_1} \right) = \left( {{\it{\varphi }}_2 - {\it{\varphi }}_1} \right)e^{ik_za}$. Hence, the zero-frequency mode must lie at **k** = 0. We then consider the wire metamaterial shown in Fig. 1c. The radii of the horizontal thick wire and the two oblique thin wires are 0.1*a* and 0.03*a*, respectively. As two meshes are interpenetrating and have independent potentials *φ*_1_ and *φ*_2_, the system has one zero-frequency mode and a similar analysis (see Supplementary Note 1 and Supplementary Fig. 1) shows that the nontrivial zero-frequency mode is at the *H* point, consistent with the band structure in Fig. 1f.

The above analysis holds regardless of the detailed geometry of the wires as long as the two meshes do not make contact. The structural details (such as the filling ratio) of the wire meshes will not affect the **k** of these zero-frequency modes but will affect the group velocities of the propagating modes emerging from these zero-frequency **k**-points. Similar argument shows that metamaterials composed of *N* interpenetrating wire meshes have (*N*-1) zero-frequency modes and the locations of these modes in **k**-space are determined solely by the connectivity of the meshes.

### Effective gauge potential in one-dimensional wire bundles

**Fig. 2:**
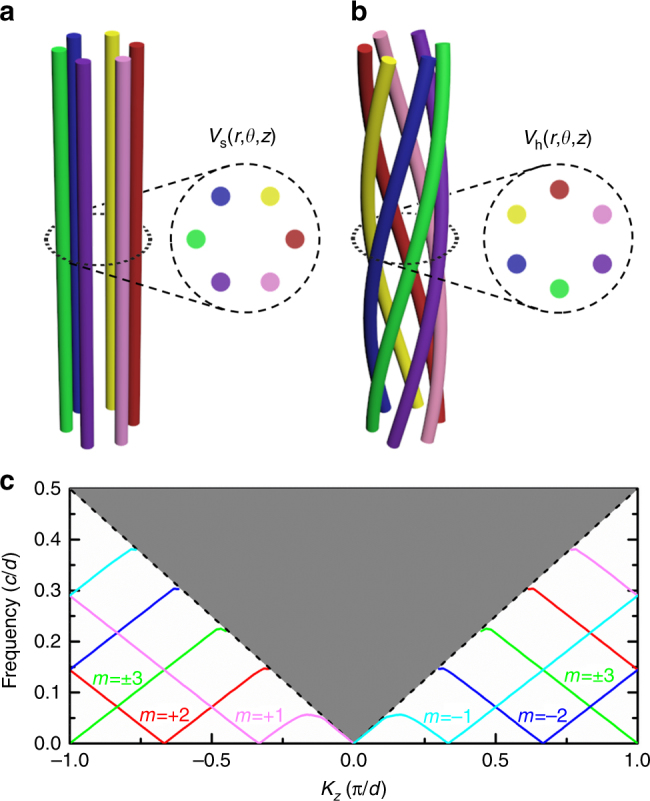
Canonical momentum generated by twisting. **a** Schematic of a 1D wire bundle. **b** Schematic of a helical wire bundle. **c** Dispersion diagram of the helical bundle shown in **b**. The gray area denotes the projected light cone. The colored lines highlight the modes propagating along the wires. The different colors indicate modes with different angular momentum *m*. These modes have an angular momentum-dependent shift in *k*_*z*_

Although the Poisson equation analysis gives the position of zero-frequency modes in **k**-space, we still need to explain under what conditions index ellipsoids can form around these zero-frequency solutions. On the other hand, it is well known that the structural modification of graphite can lead to the shifting of Dirac cone in **k**-space, which can be interpreted as a gauge potential^41–44^. To see how equifrequency surfaces can be shifted away from **k** = 0, let us consider a wire bundle composed of six straight wires aligned along the *z*-direction, as shown in Fig. 2a. The wires align on a circle with radius of *R* as shown in the inset, which shows a cross-sectional view. Under the sheath approximation^45^, the potential takes the form

1 $$V_{\mathrm{s}}\left( {r,\theta ,z} \right) = \left\{ {\begin{array}{*{20}{c}} {V_m\left( {r/R} \right)^{\left| m \right|}e^{im{\it{\theta }}},{\kern 1pt} r < R} \\ {V_m\left( {r/R} \right)^{ - \left| m \right|}e^{im{\it{\theta }}},r > R} \end{array}} \right.{\kern 1pt} \quad m = \pm 1, \pm 2...,$$

where *m* is the orbital angular momentum index. Here we take the potential at the origin to be zero. Solution with *m* = 0 are omitted, as it implies equal potentials on all wires and hence zero electric field everywhere.

Let us now twist the wire bundle, as shown in Fig. 2b. The solution of the Poisson equation becomes the superposition of

2 $$V_h\left( {r,{\it{\theta }},z} \right) = \left\{ {\begin{array}{*{20}{c}} {V_m\frac{{I_{\it{m}}\left( {m{\it{\beta }}r} \right)}}{{I_{\it{m}}\left( {m{\it{\beta }}R} \right)}}e^{im\theta }e^{ - im{\it{\beta }}z},\,\,\,\,\,r < R} \\ {V_m\frac{{K_{\it{m}}\left( {m{\it{\beta }}r} \right)}}{{K_{\it{m}}\left( {m{\it{\beta }}R} \right)}}e^{im\theta }e^{ - im{\it{\beta }}z},{\kern 1pt} \,\,\,\,\,r > R} \end{array}} \right.\quad m = \pm 1, \pm 2...,$$

where *β* = 2*π*/6*d* is the twisting ratio, *d* is the period in the *z*-direction, and *I*_*m*_ and *K*_*m*_ are the first and second kinds of the modified Bessel functions. In the limit $m{\it{\beta }}R < < 1$, Eq. (2) reduces to

3 $$V_{\mathrm{h}}\left( {r,{\it{\theta }},z} \right) \approx \left\{ {\begin{array}{*{20}{c}} {V_m\left( {r/R} \right)^{\left| m \right|}e^{im{\it{\theta }}}e^{ - im{\it{\beta }}z},\,\,\,\,{\kern 1pt} r < R} \\ {V_m\left( {r/R} \right)^{ - \left| m \right|}e^{im{\it{\theta }}}e^{ - im{\it{\beta }}z},\,\,\,\,r > \,{\mathrm{and}}\sim R} \end{array}} \right.{\kern 1pt} {\kern 1pt} \,\,\,\,\,\,\,\,\,m = \pm 1, \pm 2...$$

which has the same form as the potential of the straight wires in the region close to the wire bundle but with an additional phase factor *e*^−*imβz*^ for each angular momentum channel. This phase factor can be interpreted as the consequence of an effective gauge potential that shifts the quasistatic mode in **k** space by $\tilde k_z = - m\beta = - m\,\frac{\pi }{{3d}}$ in the twisting direction. The shift depends on the angular momentum index *m* of the quasistatic mode. We can arrive at the same conclusion using the aforementioned quasistatic consideration when the twisting ratio *β* is not small (see Supplementary Note 2 and Supplementary Fig. 2). This angular momentum-dependent shift can be confirmed by examining the calculated (using COMSOL) dispersion relation of the helical wire bundle shown in Fig. 2c. The helical wires, each of radius 0.1*d*, lie on a circle with a radius of 0.5*d*. Several guided modes (colored solid lines) emerge under the projected light cone (gray area), each labeled by its angular momentum index *m*. Zero-frequency modes appear at − *mπ*/3*d*. The C_6_ symmetry of this system implies that the modes with *m* = + 3 and *m* = − 3 share the same representation and hence the system supports five zero-frequency modes. At low frequencies, these modes exhibit a linear dispersion, where the wave speed *v* is slightly less than the wave speed in vacuum. This shifting effect can also be viewed as an analog of the rotational Doppler effect^46^ (see Supplementary Note 3).

### Forming closed equifrequency surfaces

**Fig. 3:**
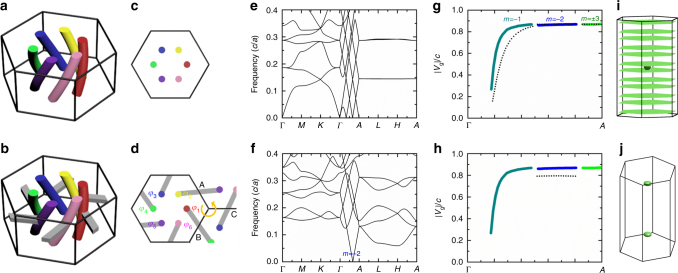
Selection of modes with different angular momenta through the introduction of in-plane connection. **a** Hexagonal array of isolated helical wire bundles. **b** Wire bundles (colored) with in-plane connecting bars (gray) that pass through the unit cell corner. The colors in **a** and **b** just highlight different helical wires. These helical wires do not necessarily have independent potentials, which depend on the connection configuration. **c**, **d** Corresponding cross-sectional views. **e**, **f** Band structures. **g**, **h** Group velocities of the linear bands emerging from the quasistatic modes (black dotted lines). For comparison, the group velocities of the guided modes in helical wire bundle shown in Fig. 2c are plotted using colored lines. Colors indicate different angular momentum *m*. **i**, **j** Equifrequency surfaces showing index ellipsoids or planes at different **k**-points. For the structure in **a**, as the wire bundles in different unit cells are isolated, the system has an infinite number of independent potentials and thus an infinite number of quasistatic modes with arbitrary (*k*_*x*_, *k*_*y*_) and discretized *k*_*z*_ = − *mβ*. These quasistatic modes can be interpreted as transmission-line modes propagating in the *z*-direction, corresponding to the flat surfaces in **i**. After introducing an in-plane connection, the system has a finite degree of freedom and forms index ellipsoids at specific **k**-points depending on the connection configuration. The in-plane connection in **b** filtrates out the quasistatic modes with *m* = ± 1, ± 3, leaving two index ellipsoids located at $$(\begin{array}{*{20}{c}} 0 & 0 & { \pm 2\pi /3d} \end{array})$$, see (**j**)

The twisting of a single bundle in real space in the *z*-direction shifts the zero-frequency solution along *k*_*z*_ and generates modes with linear dispersion along *k*_*z*_. However, we do not have a closed equifrequency surface, which requires a three-dimensional (3D) periodic structure. As such, we construct a 3D wire metamaterial by arranging helical wire^36–38^ bundles in a two-dimensional (2D) hexagonal lattice with a lattice constant of *a* in the *x*–*y* plane as shown in Fig. 3a. The calculated band structure is shown in Fig. 3e. As expected, this bundle array supports quasistatic modes locating at $\widetilde k_z = - m\pi /3d$ for *m* = ± 1,±2, ± 3. In the vicinity of $\widetilde k_z$, linear bands emerges along the *Γ*-*A* direction as shown in Fig. 3e. Their group velocities are plotted in Fig. 3g by black dotted lines, which are almost the same as that of a single bundle in Fig. 2c (colored lines in Fig. 3g). There is weak coupling between neighboring bundles since the eigenfields of the quasistatic modes localize strongly between the wires of the same bundle. The group velocities of *m* = ± 1 mode deviates from the single bundle case more than that of *m*= ± 2, ± 3, because the quasistatic mode with smaller *m* decays more slowly according to Eq. (3). Due to the weak coupling, the dispersion is nearly flat in the *k*_*x*_–*k*_*y*_ plane for any value of *k*_*z*_. In other words, the band dispersions along the *k*_*z*_ direction are almost independent of *k*_*x*_ and *k*_*y*_ (see Supplementary Note 4 and Supplementary Fig. 3). Similar flat equifrequency surfaces have been found in 2D wire arrays^39,40^, which can be used for subwavelength imaging. Apart from the linear bands emerging at $\widetilde k_z = - m\pi /3d$ (*m* = ± 1, ± 2, ± 3), two linear bands emerge from the *Γ* point (the lowest two bands from *k*_*z*_ = 0 to *k*_*z*_ = 0.1*π*/*d*), whose eigenfields resemble the plane-wave solutions with circular polarization (see Supplementary Fig. 4). As a chiral hyperbolic medium^47^, its equifrequency surface consists of an ellipse and two flat sheets centered at the *Γ* point (see Fig. 3i for equifrequency surfaces at frequency 0.02*c*/*d*). The other 10 flat sheets in Fig. 3i stem from the quasistatic modes at $\widetilde k_z = - m\pi /3d$ (*m* = ± 1, ± 2, ± 3).

In order to form index ellipsoids at nonzero **k**-points, we need to introduce connections between different wire bundles so that the system will have a finite number of independent potentials and a finite number of quasistatic modes at specific **k**-points. In the structure shown in Fig. 3b, we add six horizontal metallic bars (gray), see also the cross-sectional view in Fig. 3d. The potentials of the 6 bars are *φ*_1_ to *φ*_6_. The corresponding band structure calculated by COMSOL is plotted in Fig. 3f. Comparing the results in Figs. 3f and 2c, one finds that the 3D metamaterial in Fig. 3f only supports quasistatic modes at *k*_*z*_ = ± 2*π*/3*d* rather than five quasistatic modes in the one-dimensional bundle shown Fig. 2c. This is due to the periodic boundary condition imposed by the horizontal metallic bars on any pair of wires related by inversion symmetry (e.g., the red and green wires are connected via Bloch boundary condition). This in-plane connection imposes further requirements on the Bloch **k** and the angular momentum *m*, forbidding those with *m* = ± 1, ±3. For example, there are three metallic bars (labeled as A, B and C in Fig. 3d) encircling the right corner of the hexagonal cell marked by the solid line in Fig. 3d. The potential difference between bars A and B is (*φ*_2_ − *φ*_1_). By applying Bloch theorem, the potential difference between B and C is $\left( {{\it{\varphi }}_4 - {\it{\varphi }}_3} \right)e^{i\left( {k_x \cdot \frac{{\sqrt 3 }}{2}a - k_y \cdot \frac{a}{2}} \right)}$ and the one between C and A $\left( {{\it{\varphi }}_6 - {\it{\varphi }}_5} \right)e^{i\left( {k_x \cdot \frac{{\sqrt 3 }}{2}a + k_y \cdot \frac{a}{2}} \right)}$. By encircling the right corner in counterclockwise manner, the total potential drop is zero. Then we have $\left( {{\it{\varphi }}_2 - {\it{\varphi }}_1} \right) + \left( {{\it{\varphi }}_4 - {\it{\varphi }}_3} \right)e^{i\left( {k_x \cdot \frac{{\sqrt 3 }}{2}a - k_y \cdot \frac{a}{2}} \right)} + \left( {{\it{\varphi }}_6 - {\it{\varphi }}_5} \right)e^{i\left( {k_x \cdot \frac{{\sqrt 3 }}{2}a + k_y \cdot \frac{a}{2}} \right)} = 0$. Likewise, for the left corner of the unit cell, we have a similar equation. Meanwhile, by rotational symmetry, $\left( {{\it{\varphi }}_{j + 1} - {\it{\varphi }}_j} \right)/\left( {{\it{\varphi }}_j - {\it{\varphi }}_{j - 1}} \right) = e^{im \cdot \frac{\pi }{3}}$. Only *m* = ± 2 and *k*_*x*_ = *k*_*y*_ = 0 satisfies these requirements. According to the above discussion about wire bundles, the *k*_*z*_ component of the quasistatic modes with *m* = ± 2 should be $- m{\it{\beta }} = \mp 2\pi /3d$, giving us two index ellipsoids near $$(\begin{array}{*{20}{c}} 0 & 0 & { \pm 2\pi /3} \end{array}d)$$ in Fig. 3j. The in-plane connection also changes the group velocity of the linear band (see Fig. 3h). The **k**-points of these quasistatic modes depend only on the connectivity and are not affected by the detailed geometry of the metamaterials (see Supplementary Fig. 5). In fact, the number of quasistatic modes is determined by the degree of freedom of independent potential (see Supplementary Note 5 and Supplementary Fig. 6). We can select or exclude other quasistatic modes by forming other in-plane connections (see Supplementary Note 6 and Supplementary Fig. 7 for other examples).

**Fig. 4:**
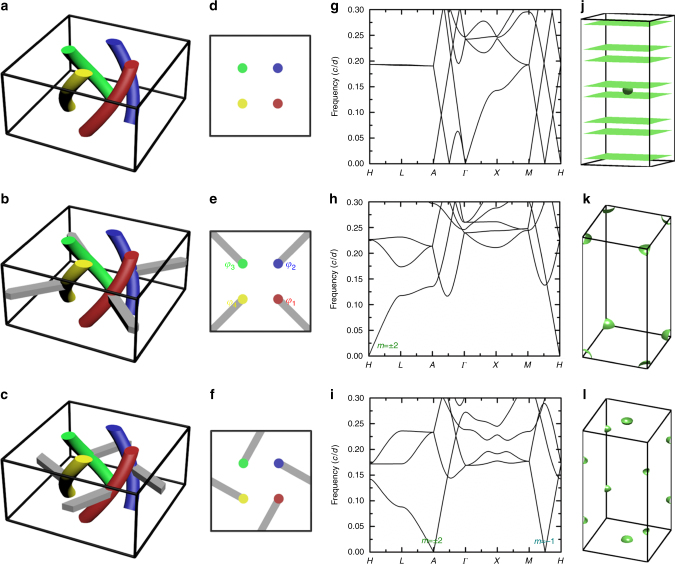
Shifting of the zero-frequency modes in the *k*_*x*_ and *k*_*y*_ directions through the introduction of in-plane connection. **a** Square array of isolated wire bundles. **b** Wire bundles (colored) with in-plane connecting bars (gray) that pass through the unit cell corner. **c** Wire bundles with in-plane connecting bars that pass through the unit cell boundary. **d**–**f** Corresponding cross-sectional views. **g**–**i** Band structures. **j**–**l** Equifrequency surfaces at 0.04*c*/*d* showing index ellipsoids or planes at different **k**-points. The in-plane connection in **b** shifts the zero-frequency mode with *m* = ± 2 to (*k*_*x*_, *k*_*y*_) = (*π*/*a*, *π*/*a*), whereas the connection in **c** maintains the *m* = ± 2 mode at (*k*_*x*_, *k*_*y*_) = (0, 0) but shifts the *m* = ± 1 modes to (*k*_*x*_, *k*_*y*_) = (*π*/*a*, *π*/*a*)

The in-plane connection can also shift the in-plane *k*_*x*_ and *k*_*y*_. Consider a square lattice of helical wires (Fig. 4) as an example. The lattice constants in the *x*-, *y*- and *z*-directions are *a* = 2*d* and *d*. The isolated bundles (Fig. 4a) have flat equifrequency surfaces generated by quasistatic modes at $k_z = - m{\it{\beta }} = - m\pi /2d$ (see the band structure and equifrequency surface in Figs. 4g and 4j). In Fig. 4b, we add four horizontal metallic bars (gray) across the unit cell corners. The Bloch condition requires that $\left( {{\it{\varphi }}_2 - {\it{\varphi }}_3} \right) = \left( {{\it{\varphi }}_1 - {\it{\varphi }}_4} \right)e^{ik_y \cdot a}$ and $\left( {{\it{\varphi }}_2 - {\it{\varphi }}_1} \right) = \left( {{\it{\varphi }}_3 - {\it{\varphi }}_4} \right)e^{ik_x \cdot a}$. Combining this with the angular momentum conditions $\left( {{\it{\varphi }}_{j + 1} - \varphi _j} \right)/\left( {\varphi _j - \varphi _{j - 1}} \right) = e^{im \cdot \frac{\pi }{2}}$, we find that *m* = ± 1 are forbidden and the only allowed solution is at *k*_*x*_ = *k*_*y*_ = *π*/*a* when *m* = ± 2. This corresponds to the light cone at the *H* point in the calculated band structure in Fig. 4h. Figure 4k plots the equifrequency surface at 0.04*c*/*d*, which consists of eight spherical surfaces at Brillouin zone corners. Figure 4c illustrates another configuration of in-plane connection. In this case, the “light cone” with *m* = ± 2 remains at $$\left( {\begin{array}{*{20}{c}} 0 & 0 & {\pi /d} \end{array}} \right)$$, whereas the “light cones” with *m* = ± 1 shift to the zone boundary $$\left( {\begin{array}{*{20}{c}} {\pi /a} & {\pi /a} & { \mp \pi /2d} \end{array}} \right)$$. The distribution of index ellipsoids is shown in Fig. 4l.

The number and positions of index ellipsoids can hence be designed using two steps: (i) use wire bundles to form an array and twist each bundle to give multiple quasistatic solutions with discrete *k*_*z*_ = − *mβ*; (ii) build in-plane connections to form closed equifrequency surfaces and to remove unwanted modes and to shift the centers of selected index ellipsoids in the *k*_*x*_–*k*_*y*_ plane. There are other ways to design these wire metamaterials, such as the structure in Fig. 1c. The connectivity of the network controls the number of index ellipsoids and their position in momentum space.

### Orientation-dependent coupling effect

The unusual quasistatic modes at nonzero **k**-points can be used to control the wave propagation using a compact structure, with a small total device size compared to the wavelength. For example, such metamaterials have an orientation-dependent coupling effect when interfaced with ordinary materials that have index ellipsoids at **k** = 0 (such as air).

**Fig. 5:**
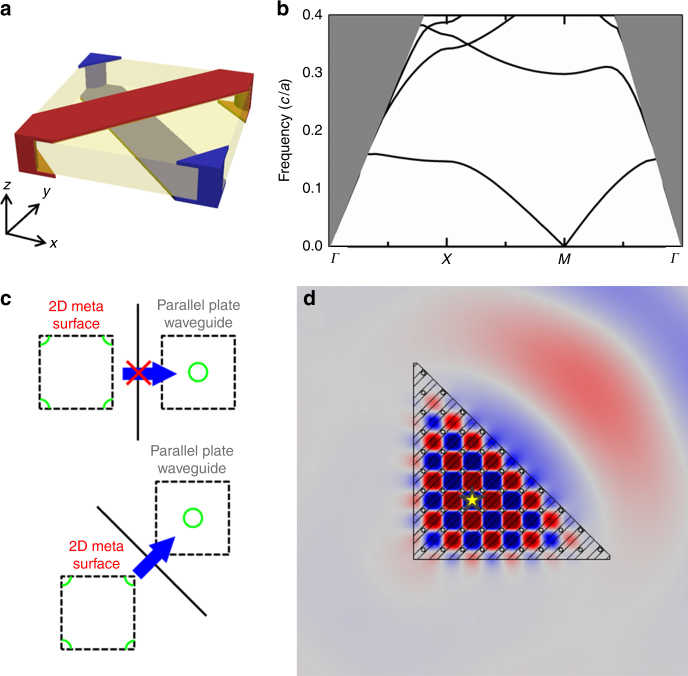
Orientation-dependent coupling between a metasurface with a shifted index ellipse and ordinary materials. **a** Square unit cell of a 2D metasurface composed of metallic stripes (blue and red) printed on both sides of a dielectric substrate with *ε* = 4 (translucent yellow region). **b** Band structure of the metasurface showing an index ellipse centered at the *M* point. The gray area is the light cone. **c** Schematics of orientation-dependent transmission between the metasurface and a parallel plate waveguide, which serves as an ordinary material with a light cone at **k** = 0. When the interface between the two materials is perpendicular to the principal axis, the waves in metasurface cannot couple to the parallel plate waveguide. The electromagnetic wave can be transmitted through the interface when the interface is tilted at 45°. **d** Simulated field pattern when a point source (yellow star) is embedded in the metasurface with a triangular boundary. The electromagnetic wave can only be coupled out through the upper right interface

These 3D metamaterials have 2D counterparts that can be implemented in the form of metasurfaces. Figure 5 shows a simple implementation, which has a square lattice with lattice constant *a*. Figure 5a shows its unit cell. The translucent yellow region is a dielectric substrate with *ε* = 4 and a thickness of 0.4*a*, and its top and bottom surfaces are decorated with two interpenetrating metallic networks (blue and red). This thin slab is surrounded by air and we focus on the guided modes in the slab. The calculated dispersion is shown in Fig. 5b where the gray region marks the light cone. An index ellipse emerges from the *M* point. This structure can be viewed as groups of twisted wires along both *x*- and *y*-directions, causing a shift of the ‘light cone’ in the *x*- and *y*-direction (see Supplementary Fig. 8). Although the wires in neighboring unit cells have different potentials, Fig. 5a is the primitive unit cell because the unit cell is determined by the structure’s geometry rather than its potential. In addition, by calculating the Fourier spectra of the eigenfields, we confirm that the index ellipse is indeed centered at a nonzero **k**-point. (see Supplementary Note 7 and Supplementary Fig. 9).

We consider wave propagation when this 2D metasurface forms an interface with an ordinary material which has index ellipse at **k** = 0, say, e.g., a parallel plate waveguide with a thickness of 0.4*a* and *ε* = 4. Schematics of the index ellipses are shown in Fig. 5c. If the interface is normal to the *x*-axis, the conservation of the **k**-component along the interface forbids the coupling of propagating modes in the metasurface to the parallel plate waveguide. If the interface is tilted at 45°, the equifrequency contours (green curves) of the two materials share a common *k*_*//*_, enabling transmission across the interface. To demonstrate this effect, we simulate a triangular slab which is a square metasurface truncated along the 45° direction (Fig. 5d). The side length of the metasurface is 10*a* (~ half wavelength). The outside region of the metasurface is the parallel plate waveguide with the same thickness. An *E*_*z*_-polarized point source (marked by a yellow star) with a frequency of 0.05*c*/*a* can only radiate through the tilted interface on the upper right as shown in Fig. 5d. Although the 2D example is purposely designed for the ease of numerical simulation, similar orientation-dependent coupling phenomena are expected for 3D wire metamaterials. The exotic index ellipsoids can be used to manipulate wave propagation even when the wavelength is much larger than the structure. In addition, such orientation-dependent coupling is a broadband effect as it depends on topology rather than on resonance. As such, the metasurface shown in Fig. 5d can serve as a broadband and compact directional antenna. Compact antenna, such as Yagi-Uda antenna, usually has a narrow bandwidth, whereas those with a broad bandwidth typically have a larger size especially for low frequencies. We simulate the far field radiation pattern in Supplementary Fig. 10a. It shows good directionality in a wide frequency range from 0.04 to 0.075*c*/*a* (see also the forward-to-back ratio in Supplementary Fig. 10b).

### Microwave experiment

**Fig. 6:**
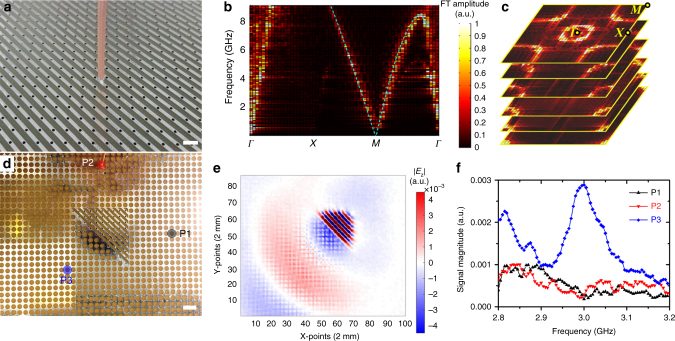
Experimental results at microwave frequencies. **a** A square sample of the metasurface with 40 × 40 periods. Scale bar, 5 mm. A source antenna was placed below the PCB board to excite the guided mode and the *E*_*z*_ field was measured by scanning a monopole antenna on top of the PCB board. **b** Fourier spectrum of the measured *E*_*z*_ field along high symmetry **k**-lines. A light cone at the M point was observed, in good agreement with the calculated dispersion (cyan dashed line). **c** Fourier spectra for different frequencies, 0.95, 1.96, 3.08, 4.42, 5.89, and 7.07 GHz (from bottom to top). The equifrequency contours form rings near the M points with increasing radii as frequency increases. **d** A top view of another sample demonstrating the orientation-dependent coupling effect, where three specific positions are labeled with color symbols (black, red and blue) and the probing antenna is located on one of them. Scale bar, 1 cm. **e** The distribution of the real part of *E*_*z*_ at frequency 2.99 GHz. **f** The signals received at three positions, as denoted by the color labels (black for P1, red for P2, and blue for P3) in **e**

We made a square sample with 40 periods in both *x*- and *y*-directions using printed circuit board (PCB) (Fig. 6a). We measured the band dispersion by exciting the guided modes using a coaxial cable below the PCB, and the *E*_*z*_ field just above the PCB was measured using a monopole antenna (see Methods). Figure 6b shows the Fourier spectrum along high symmetry lines. A light cone emerges at the *M* point, in excellent agreement with the calculated dispersion (cyan dashed line). The Fourier components near the *Γ* point stem from the propagating modes coming from the light cone in air since the *E*_*z*_ fields are measured outside the PCB. We note that the vacuum light cone does not appear in Fig. 5c, which considers the optics of the guided modes inside the slab but the light cone shows up in the measurement because the probing antenna is placed outside the slab. Figure 6c plots the Fourier spectra at different frequencies. The equifrequency contours form rings near the *M* point as predicted, with the radius increasing as the frequency increases.

We then demonstrate the orientation-dependent coupling with a triangular sample (Fig. 6d). The triangular metasurface is surrounded by a parallel plate waveguide, the bottom of which is a ground plane, while the top is a copper foil perforated with a square array of holes (lattice constant = 5 mm) where the microwave guided in the waveguide can leak out for measurement. We excite the metasurface with a coaxial cable below the PCB, and the electric field just above the PCB was measured using the monopole antenna (see Methods). Figure 6e shows the scanned field pattern (real part of *E*_*z*_) at 2.99 GHz. We observed that the wave coupled out primarily through the tilted interface, which agrees well with the simulation. The small discrepancy between Figs. 5d and 6e is because the electric fields are measured above (not inside) the PCB. Figure 6f shows the measured *E*_*z*_ at three holes (red, blue and black in Fig. 6d) in different directions from the metasurface as a function of frequency. The signal detected at the blue point is larger than those at other points, demonstrating the directivity of the radiated wave. The weak signals recorded at the black and red points are due to evanescent waves.

### Broadband-negative group velocity medium

**Fig. 7:**
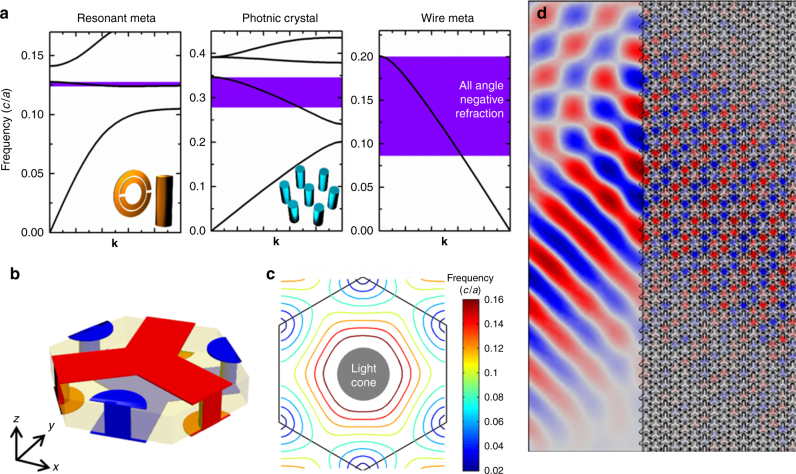
Broadband-negative group velocity medium. **a** Prototypical dispersions of resonant metamaterials, photonic crystals and wire metamaterials. The negative index of resonant metamaterials (in this case a split-ring resonator [Ref. 3]) relies on the built-in local resonance and thus works in a narrow frequency range. For photonic crystals [see, e.g., Ref. 50], the negative group velocities appear in the second or higher band and have a limited bandwidth. For metamaterials with bands emerging from nonzero **k**-points, even the lowest band can exhibit negative group velocity and have broad bandwidth. **b** Unit cell of a 2D hexagonal metasurface with negative index. **c** Equifrequency contours of the guided modes in the metasurface. At very low frequencies, the equifrequency contours are six ellipses emerging from the Brillouin zone corners. As frequency increases, these ellipses grow bigger to form a closed contour centered at *Γ* point from 0.1 to 0.17 *c*/*a* with the contour shrinks toward *Γ*, giving negative group velocities in this frequency regime. The gray solid circle marks the projected light cone. **d** Simulated field pattern when a plane wave is impinged onto the metasurface from an ordinary material (parallel plate waveguide). Negative refraction is evident. The Gaussian beam with frequency of 0.13 *c*/*a* is incident from the lower left with an angle of 45°

The light cones emerging from nonzero **k**-points give us the unique opportunity to control the refraction at low frequency (e.g., ~ 0.1*c*/*a*). By controlling the number and the position of the quasistatic modes and the slopes of the emerging linear bands (see Supplementary Fig. 11), we have many degrees of freedom to engineer the shape of equifrequency surface (see, e.g., a triangular contour in Supplementary Note 8 and Supplementary Fig. 12) and hence the refraction property in low frequency regime can be manipulated. For example, we can achieve broadband-negative group velocity medium. We note that negative refraction can be realized in metamaterials^3,7,9,10^ using resonance and photonic crystals^48–50^ using band folding. Resonant metamaterials only work in a narrow frequency range (see the schematic dispersion in Fig. 7a for a typical design^3^). For photonic crystals, the negative group velocity band is typically a higher frequency band folded back into the primitive Brillouin zone and their high central frequency limits their working bandwidth and an effective medium description is sometimes questionable if the operational frequency is high. In the wire metamaterials, the lowest frequency band emerges from nonzero **k**-points, enabling negative dispersion at low frequency (~ 0.1*c*/*a*). Our metamaterials have a broader bandwidth than the usual schemes. The effect can be exemplified using a hexagonal wire metasurface composing of three interpenetrating networks (Fig. 7b, with more details in Supplementary Fig. 13). It has two quasistatic modes at *K* and *K*’, which can be seen in the equifrequency contours in Fig. 7c. As frequency increases from zero, the ellipses at zone corners grow bigger and fuse to form a closed contour centered at *Γ* point (0.1*c*/*a*). From then on, the contour shrinks toward the *Γ* point, giving a negative group velocity. Negative refraction effect can be demonstrated by impinging a Gaussian beam at an angle onto the metasurface (Fig. 7d). The field pattern of the refracted beam in Fig. 7d shows negative refraction propagation consistent with the equifrequency contours in Fig. 7c. We note that this is a fairly broadband effect. Similar concepts can be extended to 3D wire metamaterials.

In addition, these connected wire mesh media can obviously be used to realize a cavity without wall (see Supplementary Note 9 and Supplementary Fig. 14).

## Discussion

We have proposed a new type of metamaterial composed of multiple interpenetrating wire meshes. The connectivity of the wire meshes can be used to control the number and position of the index ellipsoids and the light cones emerging from nonzero **k**-points can be used to generate exotic equifrequency surfaces at low frequencies. The unusual equifrequency surfaces of such medium can provide a new platform to provide broadband functionality such as orientation-dependent coupling effect or broadband-negative refraction, and can be utilized to manipulate the electromagnetic wave using subwavelength structures. The broadband functionality is a consequence of connectivity rather than resonance. Although some wire media with index ellipsoids at **k** = 0 can be described using homogenization theories^27,29,30^, it will be challenging to find an effective medium description for these materials with index ellipsoids at nonzero **k**-points.

## Methods

### Experiment demonstrating the band dispersion

The sample was fabricated on a PCB with dimensions of 40*a* × 40*a*. The lattice constant was 5 mm and the thickness of the PCB was 2 mm. The middle layer is the PCB dielectric. A coaxial cable was connected with the metasurface, with its inner and outer conductor soldered on the two set of wires of the metasurface respectively to feed in microwave signals. The soldering was done on the bottom surface. Another coaxial cable with its inner conductor extruded was mounted vertically above the sample to probe the surface field *E*_*z*_. The cables were wired with a microwave network analyzer (Agilent N5230C), and the S21 parameter (magnitude and phase) was recorded. In the experiment, the probing cable (monopole antenna) is movable via a 2D translational stage to receive the microwave at specific positions and to map the *E*_*z*_ field in scanning mode.

### Experiment demonstrating the orientation-dependent coupling

The sample is designed to have a triangular metasurface surrounded by parallel plate waveguide, where the bottom plate is a ground plane and the top plate is patterned with a square array of holes (lattice constant = 5 mm) to allow the wave to leak out for measurement. A coaxial cable was connected with the metasurface from the bottom surface to feed in microwave signals. Another coaxial cable with its inner conductor extruded was mounted vertically above the sample to probe the surface field *E*_*z*_, which is leaked from the electromagnetic mode in the dielectric layer.

### Data availability

The data that support the findings of this study are available from the corresponding author on request.

## Electronic supplementary material

Supplementary Information
