# Supplementary material for: Metamaterials with index ellipsoids at arbitrary k-points
Source: Nat Commun. 2018 May 25;9:2086. doi: 10.1038/s41467-018-04490-4 (PMC5970243; doi:10.1038/s41467-018-04490-4)
Supplement: Supplementary file 1 — Supplementary Information [file 41467_2018_4490_MOESM1_ESM.pdf]

Supplementary Information

of

“Metamaterials with index ellipsoids at arbitrary k-points”

Chen et al.

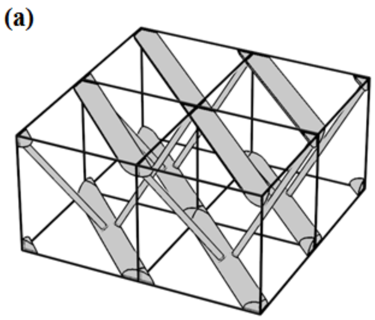

Supplementary Figure 1 | 2x2 supercell of the double wire mesh metamaterials shown in Fig. 1(c). In panel (a), all metals are shown in the same colour (grey). Panel (b) shows the same structure, but the red and blue colour illustrates the fact that the structure has two disjoint networks, with the connected parts rendered in the same colour.

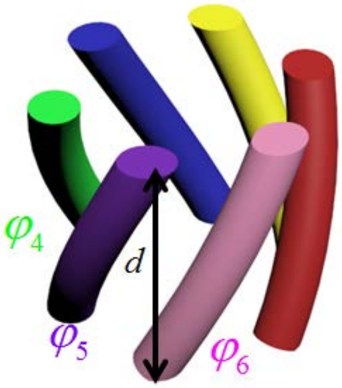

Supplementary Figure 2 | Unit cell of the single helical wire bundle. The period in *z*-direction is *d* and corresponding twisting ratio is $\beta = \pi / 3d$.

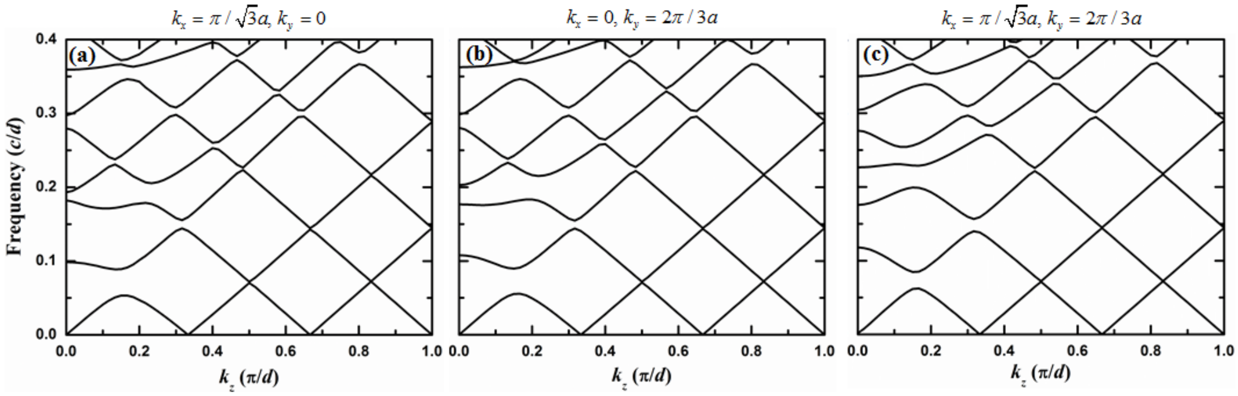

Supplementary Figure 3 | Transmission-line modes of the hexagonal array of isolated helical wire bundles. Dispersions along *z*-direction are calculated with different fixed k_x and k_y, (a)

$k_x = \pi/\sqrt{3}a, k_y = 0$, (b) $k_x = 0, k_y = 2\pi/3a$, (c) $k_x = \pi/\sqrt{3}a, k_y = 2\pi/3a$. We see that the 1D cone dispersion at quasistatic limit exists for arbitrary k_x and k_y.

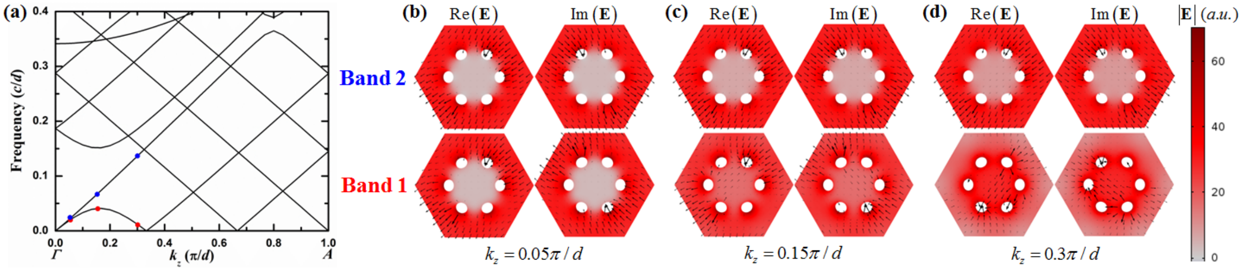

Supplementary Figure 4 | Low frequency modes propagating in the background of the hexagonal array of isolated helical wire bundles. (a) Band dispersion along the *Γ*-*A* direction. (b-d) Eigen electric field patterns of the lowest two bands at different k_z, which are highlighted by the blue and red dots in (a). Black arrows plot the electric fields while colours (white to red) plot the magnitudes |**E**|. In (b), when k_z is small ($k_z = 0.05\pi/d$), the lowest two bands resemble the plane wave modes propagating along the *z*-direction in the air background with left-handed polarization (for band 2) or right-handed polarization (for band 1). Note in particular that the real and imaginary part of the **E** field are aligned along orthogonal axes. Most of the fields localize outside the wire bundle. As k_z increases (c,d), the RCP mode begins to interact with the guided mode in the wire bundle and the electric fields come into the bundle. Thus the dispersion of band 1 exhibits avoid-crossing. On the other hand, the LCP mode has no interaction with the mode guided along the bundle since they belong to different representations (different m). Thus band 2 exhibits linear dispersion and the electric fields remain to be localized in the background.

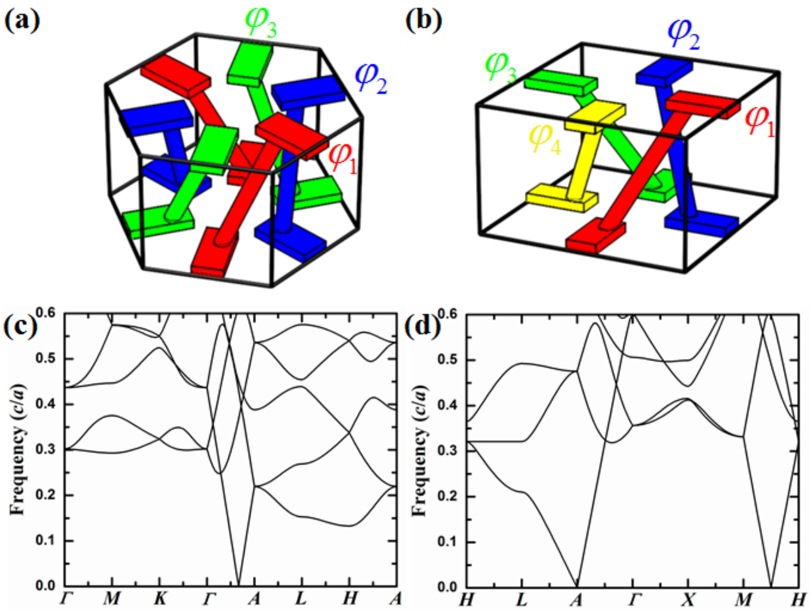

Supplementary Figure 5 | Wire metamaterials with different detailed geometry but the same connectivity. (a) Wire metamaterial with the same connectivity as that in Fig. 3b. (b) Wire metamaterial with the same connectivity as that in Fig. 4c. (c) & (d) Corresponding band structures. In both structures, the horizontal and vertical lattice constants are a and d=0.6a. The helical wires are replaced by slanted cylinders and the in-plane connecting bars are changed to have a more tabular cross section. Here different colours highlight sets of unconnected meshes with independent potentials. We see that the structure in (a) still has two light cones at $(0 \quad 0 \quad -2\pi/3d)$ and $(0 \quad 0 \quad 2\pi/3d)$ and that the structure in (b) still has three light cones at $(\pi/a \quad \pi/a \quad \pi/2d)$, $(\pi/a \quad \pi/a \quad -\pi/2d)$ and *A* point.

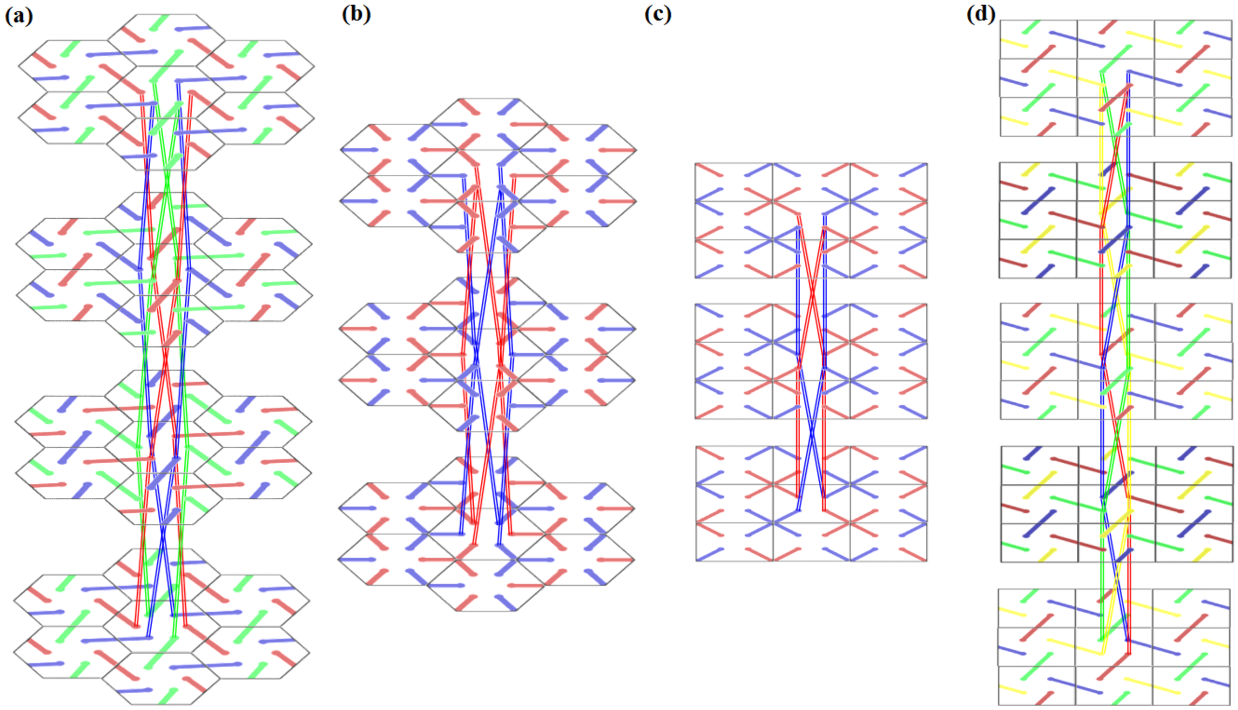

Supplementary Figure 6 | Wire metamaterials with many unit cells displayed to better illustrate the connectivity. (a) Wire metamaterial shown in Fig. 3b which consists of three sets of wire meshes. (b) Wire metamaterial shown in Fig. S7a which consists of two sets of wire meshes. (c) Wire metamaterial shown in Fig. 4b which consists of two sets of wire meshes. (d) Wire metamaterial shown in Fig. 4c which consists of four sets of wire meshes. Here different colours highlight different sets of wire meshes that have independent potentials. For clarity, only the interlayer connections (double lines) in the center unit cell are plotted. In the metamaterial, every unit cell has the same interlayer connection.

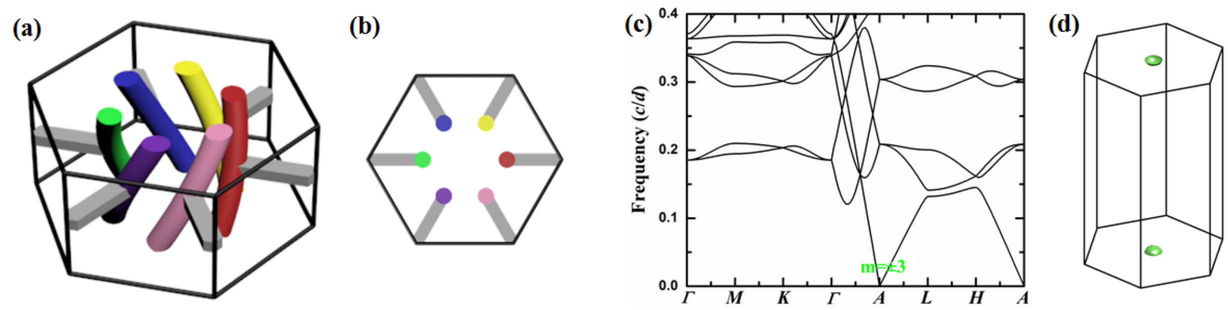

Supplementary Figure 7 | Hexagonal array of helical wire bundle that are connected by the horizontal bars across the unit cell corner. (a,b) Oblique view and cross sectional view of the unit cell. (c) Calculated band structure. (d) Equifrequency surface at 0.02*c*/*d*. This configuration filtrates out the quasistatic modes with *m*=±1,±2 leaving an index ellipsoid at Brillouin zone boundary *A*.

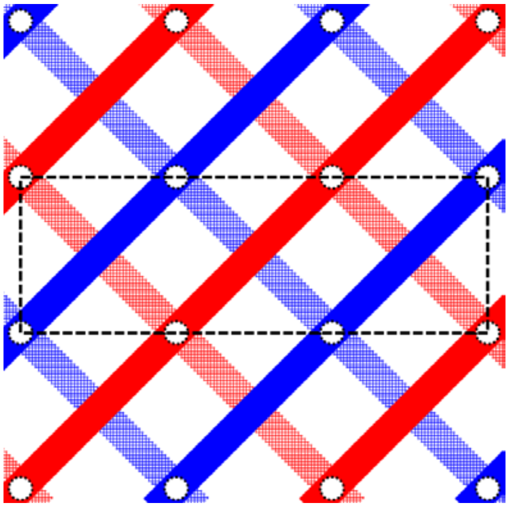

Supplementary Figure 8 | Top view of a 3x3 supercell of the metasurface. The metallic stripes on the top/bottom of the substrate are plotted in solid/shaded colours. Blue and red represent two sets of interpenetrating meshes. The white dotted circles represent the metallic vias connecting the top and bottom layers. This structure can be viewed as groups of twisted wires along both *x*- and *y*-directions. For example, the black dashed box highlights a wire bundle composed of two left-handed intertwined helical structures in the *x*-direction, causing a shift of the 'light cone' in the *x*-direction. Likewise, right-handed helical conduction pathways can be found in the *y*-direction.

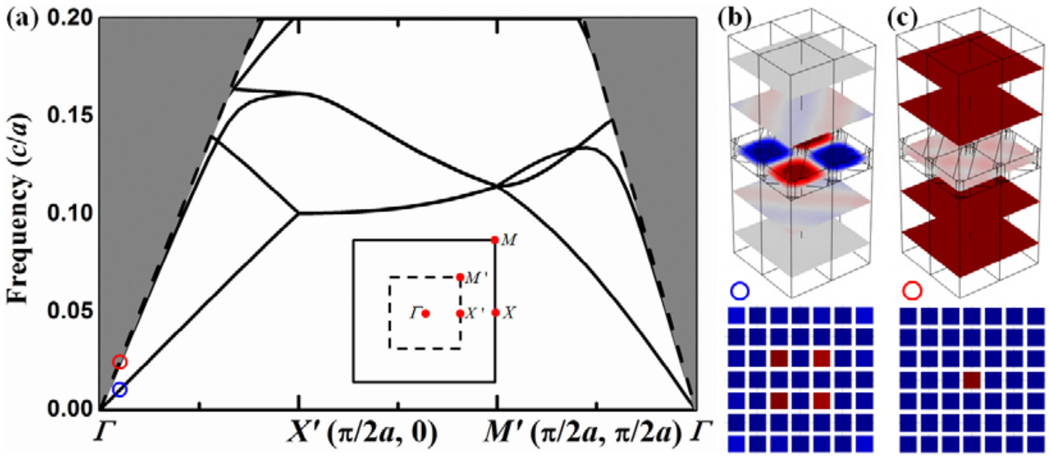

Supplementary Figure 9 | Band structure of the 2x2 supercell of the square metasurface. (a) Band dispersion along the high symmetry lines. The dashed (solid) square in the inset denotes the Brillouin zone in the supercell (unitcell) configuration. (b) E_z field of this linear band at $(k_x,k_y)=(\pi/20a,\ 0)$ which corresponds to the blue open circle in (a). The fields at five cut planes with z=-2a,-a,0,a,2a are shown. Lower panel shows the amplitudes of its Fourier components. Dark blue represents zero while red represents positive number. The centered patch of the 7x7 array shows the amplitude of **k**-component with $(k_x,k_y)=(\pi/20a,\ 0)$ in the first Brillouin zone. We can see that the Fourier components have strong amplitudes on the four patches with $(k_x,k_y)=(\pi/20a\pm\ \pi/a,\ \pm\ \pi/a)$ in the extended Brillouin zone. It indicates that this mode should be an eigen mode near the M point in the original (unitcell) configuration. (c) E_z field of the propagating in air which corresponds to the red open circle in (a). For reference, lower panel plots the amplitudes of its Fourier components and most of the Fourier components concentrate in the first Brillouin zone.

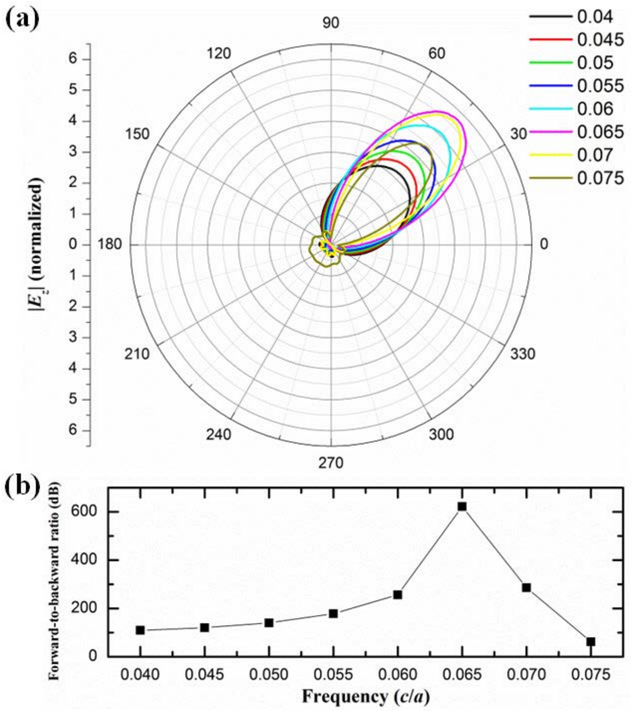

Supplementary Figure 10 | Broadband directionality of the triangle metasurface shown in Fig. 5d. (a) Far field radiation patterns for different frequencies. (b) Forward-to-backward ratio between the main lobe and the rear lobe of the far field radiation. The ratio has a peak near 0.065*c/a* due to the minima of the rear lobe.

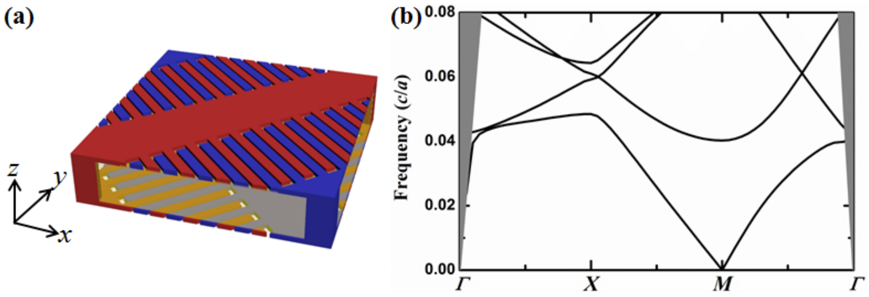

Supplementary Figure 11 | Tuning the group velocity. (a) The metasurface has the same connectivity as that shown in Fig. 5a except that additional protrusions extend from the original metallic stripes to increase capacitance. (b) Corresponding band structure. The increased capacitance between the two interpenetrating networks slows down the group velocity to ~0.1 times the speed of light in vacuum. The group velocity can be even slower by introducing denser groove structures.

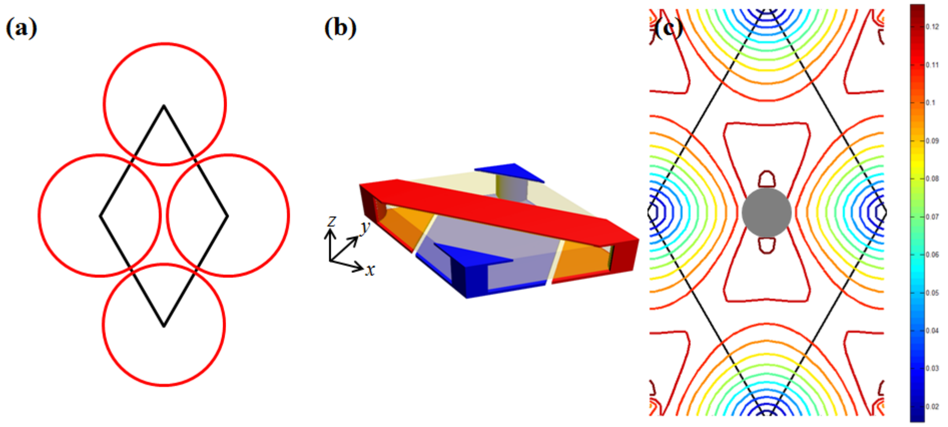

Supplementary Figure 12 | Engineering the shape of equifrequency contour of the lowest band. (a) Schematic showing that a triangular equifrequency contour can form when three index ellipses emerging from 3 **k**-points are converging. (b) A 2D metasurface with a rhombic lattice. (c) Corresponding equifrequency contours for the lowest band of the guided modes. The contour with frequency of 0.11 *c/a* forms two triangles (parts of which are shadowed by the projected light cone (gray solid circle) as the slab is bounded by free space above and below.

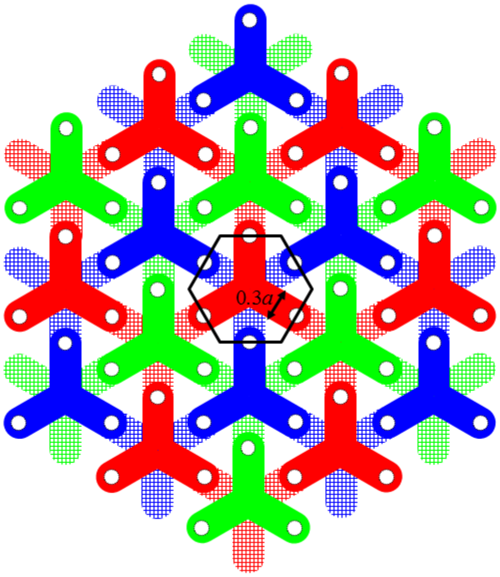

Supplementary Figure 13 | Top view of a supercell of the hexagonal metasurface shown in Fig. 7b. The metasurface is composed of three interpenetrating networks (red, blue and green). The Y-shaped metallic patches on the top/bottom of the substrate are plotted in solid/shaded colours. The widths of metallic stripes are 0.3*a*, where *a* is the lattice constant. The thickness of the dielectric substrate is 0.2 *a*.

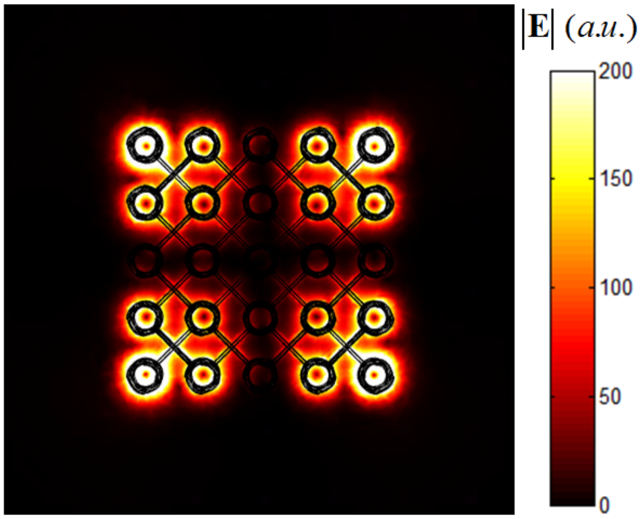

Supplementary Figure 14 | Cavity without wall. Electric field profile of the cavity mode at 0.0304 *c/d* when the wire metamaterial shown in Fig. 4b with 5 x 5 x 9 unit cells (10*d* x 10*d* x 9*d*) surrounded by air.

Supplementary Note 1: Quasistatic potential analysis of the double wire mesh structure shown in Fig. 1c

To see the connectivity of the two meshes, we plot a 2x2 supercell in Supplementary Fig. 1. In panel (a), all metals are shown in the same colour (grey). Panel (b) shows the same structure, but the red and blue colour illustrates the fact that the structure has two disjoint networks, with the connected parts rendered in the same colour. Since the two meshes do not touch, they have independent potential φ_1 and φ_2. Considering the four corners on the top surface of the unit cell in Fig. 1c, one has $(\varphi_2-\varphi_1)=(\varphi_1-\varphi_2)e^{ik_xa}$ and $(\varphi_2-\varphi_1)=(\varphi_1-\varphi_2)e^{ik_ya}$. For the Bloch boundary in the *z*-direction linking the top surface and the bottom surface of the unit cell, we have $(\varphi_2-\varphi_1)=(\varphi_1-\varphi_2)e^{ik_za}$. Therefore $\mathbf{k}=(\pi/a \quad \pi/a \quad \pi/a)$ which is consistent with the band structure in Fig. 1f.

Supplementary Note 2: Quasistatic potential analysis of the helical wire bundle

We can understand the shift of quasistatic modes in helical wire bundle by considering the quasistatic potential. Supplementary Figure 2 shows its unit cell. Suppose the quasistatic potentials of the green, violet and pink wires are φ_4, φ_5 and φ_6. Due to the C_6 symmetry, they are related by angular momentum, $(\varphi_6 - \varphi_5) = (\varphi_5 - \varphi_4)e^{im\frac{\pi}{3}}$. The top end of the violet wire and the bottom end of the magenta wire are related by a translation in z-direction (so do the top end of green wire and the bottom end of violet), thus Bloch boundary requires that $(\varphi_5 - \varphi_4) = (\varphi_6 - \varphi_5)e^{ik_z d}$. Then we have $k_z = -m\pi / 3d = -m\beta$, where the quasistatic mode with m appears. This conclusion applies even when the twisting ratio is not small.

Supplementary Note 3: The shift of quasistatic modes as an analogue of rotational Doppler effect

The shifting effect for a single helical wire bundle can also be viewed as an analogue of rotational Doppler effect, in which a harmonic wave with an orbital angular momentum index m will be observed at a shifted frequency $\Delta\omega = \pm m\Omega$ in a rotating system with an angular frequency Ω, where the sign depends on the direction of rotation. In our case, the twisting in the z-direction plays the analogous role of the evolution of time and the rotation of the wires along z-direction shifts the zero frequency modes in **k**-space through the relation $\tilde{k}_z = \pm m\beta$, where β plays the role of Ω.

Supplementary Note 4: The band dispersion of the hexagonal array of isolated wire bundles

In Supplementary Fig. 3, we plot the dispersions for three different choices of k_x and k_y. They look almost identical and all show 1D linear cone behaviors near $\begin{pmatrix} k_x & k_y & -m\pi / 3d \end{pmatrix}$ with $m = \pm 1, \pm 2, \pm 3$. The existence of infinite number of quasistatic modes with $\begin{pmatrix} k_x & k_y & -m\pi / 3d \end{pmatrix}$ can be understood by using quasistatic potential argument. Since the wires in neighboring unit cells do not contact with each other, all of the wires have independent potentials. Thus the

system has infinite degrees of freedom and infinite number of quasistatic modes. In fact, these modes can be regarded as transmission-line modes along each helical wire bundle.

Apart from the linear bands emerging at $\tilde{k}_z = -m\pi / 3d$ ($m = \pm 1, \pm 2, \pm 3$), two linear bands emerge from the Γ point in Supplementary Fig. 4a. Their eigen fields are plotted in Supplementary Fig. 4b, for different k_z. When k_z is small ($k_z = 0.05\pi / d$), the lowest two bands resemble the plane wave modes propagating along the z-direction in the air background with left-handed polarization (for band 2) or right-handed polarization (for band 1). Most of the fields localize outside the wire bundle. As k_z increases (Supplementary Figs. 4c,d), the RCP mode begins to interact with the guided mode in the wire bundle and the electric fields come into the bundle. Thus the dispersion of band 1 exhibits avoid-crossing. On the other hand, the LCP mode has no interaction with the mode guided along the bundle since they belong to different representations (different m). Thus band 2 exhibits linear dispersion and the electric fields remain to be localized in the background. Note that these two bands can also be found in Fig. 2c for a single bundle. One is the cyan band when k_z approaches to zero. The other is the circular polarized plane-wave propagating in the air background (the black dashed line in Fig. 2c).

Supplementary Note 5: The number of index ellipsoid and the connectivities of the wires

The number of index ellipsoid is determined by the number of independent potentials (disconnected wire meshes). We take the wire metamaterial in Fig. 3b for example. To see whether the wire meshes have independent potentials, one needs to plot the structure with many unit cells (see Supplementary Fig. 6a). Here different colours highlight different sets of wire meshes that have independent potentials. For clarity, only the interlayer connections (double lines) in the center unit cell are plotted. We found that the helical wires on opposite sides are connected and have the same potential. Thus this wire metamaterial is composed of three interpenetrating wire meshes (red, blue and green). Due to the uniqueness theorem, the system has two nontrivial and linearly independent solutions for the electric field at zero frequency. This is consistent with the computed band structure in Fig. 3f, which shows a light cone emerging from a nonsymmetric point between the Γ and the A points. The other zero frequency mode can

be inferred by applying time-reversal symmetry. To see the connectivities, we also plot the other three wire metamaterials (those in Supplementary Fig. 7a, Figs. 4b & 4c) with many unit cells in Supplementary Fig. 6. Their numbers of index ellipsoids are also consistent with the numbers of disconnected wire meshes. Similarly, we can design wire metamaterials with index ellipsoid at other **k**-point with fractional number of reciprocal lattice and can also introduce more index ellipsoids by adding more wire meshes.

Supplementary Note 6: Excluding quasistatic modes by designing the in-plane connection

In Supplementary Fig. 7, we selectively exclude the quasistatic modes with $m = \pm 1, \pm 2$ by considering another configuration of in-plane connection in hexagonal array of wire bundle. We add six metallic bars (gray) in a way shown in Supplementary Fig. 7b and impose the condition that the connected metallic helixes to have equal potentials in the zero frequency limit. For example, the pink and yellow (violet and blue) helixes in Supplementary Fig. 7b are connected by metallic bars through the unit cell boundary along the *y*-direction. Then we have $(\varphi_2 - \varphi_3) = (\varphi_6 - \varphi_5)e^{ik_y \cdot a}$. Meanwhile, $(\varphi_2 - \varphi_3) = (\varphi_6 - \varphi_5)e^{im \cdot \frac{2}{3}\pi}$. Similar relations can be found for the Bloch boundaries in the other two directions. One finds that only $m = \pm 3$ satisfies these requirements. Its band structure is calculated in Supplementary Fig. 7c demonstrating that it supports only one light cone at $\begin{pmatrix} 0 & 0 & \pi / d \end{pmatrix}$. Supplementary Figure 7d calculates its equifrequency surface at 0.02*c*/*d* (two hemispherical surfaces) indicating an index ellipsoid existing at zone boundary.

Supplementary Note 7: Band structure description using a (2x2) unit cell

We note that band structure results are typically displayed using the reduced zone scheme. If we choose a bigger unit cell, the light cone at non-zero **k**-points can be "folded back" into the Γ point within the reduced zone scheme. But the physics cannot be changed by choosing a different cell. What is important is the Fourier component of the Bloch modes. If we perform a Fourier analysis of the Bloch modes, the "physically meaningful and dominant" **k**-components (measured in absolute units) will not change if we enlarge the unit cell. For example, if we

purposely choose a bigger unit cell to fold the light cone back to zone center, the Bloch modes at low frequencies will still have predominately strong components at non-zero **k**-points.

To illustrate this point, we calculate the band structure of the square metasurface in Fig. 5a using a 2x2 supercell. Results are shown in Supplementary Fig. 9a. Dashed (solid) square in the inset shows the Brillouin zone boundary corresponding to the supercell configuration. The light cone originally lying at *M* point is folded back to the Brillouin zone center because of the larger supercell configuration. But the nonzero **k** of this index ellipse can still be verified by examining its eigen field. Supplementary Figure 9b plots the E_z field pattern of the mode that is denoted by blue open circle in Supplementary Fig. 9a. We see that most of the fields localize inside the PCB board and their phases are almost opposite in the neighboring unit cells. Lower panel calculates the amplitudes of different **k**-components of this eigen mode. The colour of the centered patch in the 7x7 array represents the amplitude with $(k_x,k_y)=(\pi/20a, 0)$. It is seen that the Fourier field components concentrates on the four **k**-components with $(k_x,k_y)=(\pi/20a\pm \pi/a, \pm \pi/a)$. This is consistent with the fact that it is an eigen mode near *M* point if we choose a primate unit cell. For comparison, Supplementary Figure 9c calculates the Fourier components of the propagating mode in air. Most of its Fourier field components concentrate in the zone center (the red patch at the center).

Supplementary Note 8: Engineering the equifrequency contour of the lowest band

The ability to control the number and position of the quasistatic modes gives us the freedom and flexibility to design the shape of the equifrequency surface at low frequency. To illustrate this idea, we consider the Brillouin zone of a 2D rhombic lattice (see for example, the black rhombus in Supplementary Fig. 12a). Such a system has one quasistatic mode locating at the zone corner. The left, right and up (down) corners of the rhombus (Brillouin zone boundary) are equidistant and form a regular triangle. The quasistatic mode at these points can be viewed as the starting points of the low frequency contour and equifrequency contours are concentric circles emerging from these corners. As frequency increases from zero, equifrequency contours will become bigger and three contours (the left, right and up circles in Supplementary Fig. 12a) will come together to form a triangle. Likewise, another triangle forms in the lower half of the Brillouin zone. Supplementary Figure 12b shows the unit cell of metasurface with a rhombic lattice. This

structure is similar to that in Fig. 5a, but its lattice is compressed along the y-direction in order to obtain the rhombic Brillouin zone in Supplementary Fig. 12a. The rhombic cell is $\sqrt{2}a$ ($\sqrt{2/3}a$)-long in the major (minor)-axis direction. Since it has the same connectivity as that in Fig. 5a, this metasurface has one quasistatic mode at zone corner. Supplementary Figure 12c calculates the equifrequency contour of the lowest band. The contour with frequency of 0.11 *c/a* forms two triangles although parts of them are shadowed by the projected light cone (gray solid circle) coming from free space above and below the meta-slab.

By introducing more quasistatic modes and controlling the slopes of the linear bands, different shapes of equifrequency contour/surface can in principal be designed to exhibit exotic refraction or wave transport behavior at low frequency. We note that complicated equifrequency surface can be obtained in the high frequency band of photonic crystals using band folding (Bragg scattering). Here we show that, with the new degree of freedom of quasistatic mode at nonzero **k**-point, exotic equifrequency surface (such as negative refraction medium) can also be obtained in low frequency and have broad bandwidth.

Supplementary Note 9: Cavity without wall

The quasistatic modes at nonzero **k**-points can be used to realize a cavity without wall. Consider a cube of the wire metamaterial shown in Fig. 4b, which has an index ellipsoid at Brillouin zone corner. When all of its interfaces are normal to the principle axis (*x*-, *y*- and *z*-direction), the EM wave propagating inside the medium can hardly couple out to the plane wave mode in air due to the mismatched $k_{//}$ component. This can serve as a cavity without wall at low frequency. Supplementary Figure 14 simulates the metamaterial with 5 x 5 x 9 unit cells (10*d* x 10*d* x 9*d*) surrounded by air. A point source with frequency of 0.0304 *c/d* is placed at $(2.5d \quad 2d \quad 0)$ to excite the cavity mode.
